# Supplementary material for: The experiences of high-risk young adults with type 1 diabetes transitioning to real-time continuous glucose monitoring – A thematic analysis
Source: PLoS One. 2025 May 2;20(5):e0320702. doi: 10.1371/journal.pone.0320702 (PMC12047766; doi:10.1371/journal.pone.0320702)
Supplement: S1 File — (DOCX) [file pone.0320702.s001.docx]

**S1: Semi-structured interview schedule for baseline and post-rtCGM interviews**

**Interview schedule – Baseline**

Thank you for agreeing to speak with me today. I expect that the interview will take about an hour, during which time I’ll be asking you some questions about your experiences. Of course, if there are any questions that you would rather not answer, that is absolutely fine. And if you wish to stop the interview at any stage, this is also fine. The interview will be audio recorded and I will later type up this conversation. But it will be anonymous because you will not be identified by name.

Sometimes people tell me things that can make me worried about them or other people, and if this happens I might need to tell someone else so that we can make sure that everyone is safe, but if that happens I would try to tell you first.

Do you have any questions before we begin?

*Background*

1. Who do you live with?
2. What do you do?

*Work / college / university / unemployed*

*Diabetes*

1. When were you diagnosed with diabetes?
2. What do you remember about the time you were diagnosed? *Depending on age at diagnosis* – How did you feel? Was there anyone who helped you with your management? What did they do?
3. How do you feel about living with diabetes? What is the impact of it on your life?

*e.g. friendships / romantic relationships / with family / work or education*

1. What is your current regime?

*CSII / MDI, monitoring, adherence and barriers*

1. *If high HbA1c* – At the moment, your HbA1c is considered to be high. What is your understanding of what that means for you? *If familiar with HbA1c* – Has it always been around this level, or has it previously been higher/lower? *If previously different* – Do you know what was different about those times?

*If DKA/hyperglycaemia admissions* – You have had some hospital admissions with DKA/high blood glucose levels. What happened? How do you feel about those times?

1. Have you ever had any hypos where you needed help from someone else? *If yes -* What happened?
2. Do you worry about having hypos? *If yes* - Does this affect how you self-manage your diabetes?
3. How do you find your diabetes clinic appointments? How often do you go to appointments? *If not attending all appointments* – What can stop you attending?
4. *If relevant -* What has it been like moving from being a child with diabetes to an adult?

*e.g. services / more responsibility for self-management / impact on diabetes self-management / managing diabetes at this life stage*

1. Are you happy with your diabetes management at the moment? Is there anything you would like to change? If so, are there any things that make those changes difficult?

*Other*

1. Is there anything that we’ve not talked about that you would like to tell me?
2. Do you have any questions for me?

**Interview schedule – post rtCGM**

Thank you for agreeing to speak with me today. I expect that the interview will take up to an hour, during which time I’ll be asking you some questions about your experiences. Of course, if there are any questions that you would rather not answer, that is absolutely fine. And if you wish to stop the interview at any stage, this is also fine. The interview will be audio recorded and I will later type up this conversation. But it will be anonymous because you will not be identified by name.

Sometimes people tell me things that can make me worried about them or other people, and if this happens I might need to tell someone else so that we can make sure that everyone is safe, but if that happens I would try to tell you first.

Do you have any questions before we begin?

*RT-CGM*

1. What did you think when you were offered the RT-CGM for six months? How did you feel about it?

*e.g. excited / annoyed at being asked to do something else / worried about the social implications*

2. Did you have any particular hopes or expectations about how it might affect your diabetes?

1. How did you use it?

*Wear time / contexts when worn and contexts when not worn / how it was used to inform self-management*

*If appropriate – ask about barriers to using it more – e.g. forgetting / bulkiness / social concerns / lifestyle concerns / constant reminder about diabetes*

1. What did you think about it overall?
2. What did other people think about it?
3. Do you think it was useful to you? *If so* – In what way? Do you think there are things that might have made it more useful?
4. Has it had any impact on how you experience living with diabetes?

*Prompts – e.g. does diabetes feel like more/less of a burden?*

1. Would you recommend RT-CGM to a friend with type 1 diabetes? Why / why not?

*Other*

9. Is there anything that we’ve not talked about that you would like to tell me?

10. Do you have any questions for me?
